# Infectious Agents and Bone Marrow Failure: A Causal or a Casual Connection?

**DOI:** 10.3389/fmed.2021.757730

**Published:** 2021-11-04

**Authors:** Valentina Giudice, Antonio M. Risitano, Carmine Selleri

**Affiliations:** ^1^Department of Medicine, Surgery and Dentistry Scuola Medica Salernitana, University of Salerno, Baronissi, Italy; ^2^Hematology and Transplant Center, University Hospital San Giovanni di Dio e Ruggi d'Aragona, Salerno, Italy; ^3^Hematology and Hematopoietic Stem Cell Transplantation Unit, AORN San Giuseppe Moscati, Avellino, Italy; ^4^Department of Medicine and Surgery, University of Naples Federico II, Naples, Italy

**Keywords:** infectious diseases, TCR repertoire, bone marrow failure, bone marrow failure syndrome, immune response

## Abstract

Acquired bone marrow failure (BMF) syndromes are considered immune-mediated disorders because hematological recovery after immunosuppressive therapies is the strongest indirect evidence of the involvement of immune cells in marrow failure development. Among pathophysiology hypotheses, immune derangement after chronic antigen exposure or cross-reactivity between viral particles and cellular components are the most accepted; however, epitopes against whom these lymphocytes are directed to remain unknown. In this study, we showed that BMF-associated immunodominant clones, namely the most represented T cells carrying an antigen-specific T-cell receptor (TCR) sequence in a random pool, were frequently associated with those described in various infectious diseases, such as cytomegalovirus (CMV) and *Mycobacterium tuberculosis* infection. We hypothesize that these pathogens might elicit an autoimmune response triggered by cross-reactivity between pathogen-related components and proteins or might be expanded as an unspecific response to a global immune dysregulation during BMF. However, those frequent intracellular pathogens might not only be passengers in marrow failure development, while playing a central role in starting the autoimmune response against hematopoietic stem cells.

## Introduction

Bone marrow failure (BMF) syndromes are a heterogeneous group of benign hematological diseases characterized by uni or multilineage marrow and/or peripheral blood cytopenia(s) (1, 3–5) and included acquired aplastic anemia (AA), hypoplastic myelodysplastic syndromes (MDS), paroxysmal nocturnal hemoglobinuria (PNH), and large granular lymphocyte (LGL) leukemia (6, 7). Patients can also display a combination of those clinical entities: small PNH clones are frequently found in AA, or MDS might arise from a long-lasting AA (6). Acquired marrow failure syndromes are considered immune-mediated disorders, and hematological recovery after immunosuppressive therapies (IST), such as cyclosporine, is the main indirect evidence of the predominant role of the immune system in marrow failure development. Despite a common pathogenetic “fil rouge,” BMF syndromes differ for clinical manifestations, molecular and cytogenetic alterations, and types of immune cells involved (8).

## Classical Pathogenetic Hypothesis

### Acquired Aplastic Anemia

Acquired AA is an immune-mediated BMF syndrome caused by an autoimmune attack against hematopoietic stem cells (HSCs) and progenitor cells leading to progressive depletion of the stem cell pool and consequent peripheral cytopenia(s) (9). The main pathogenetic hypothesis links an unknown viral infection affecting stem cells to cross-reactivity with self-antigens and subsequent autoimmune clone expansion. Infected cells show on cell surface viral particles processed and presented by antigen-presenting cells to naïve CD4^+^ T cells (10) that, once activated, preferentially differentiate in T helper (Th) 1 cells, the predominant CD4^+^ T-cell subset involved in viral clearance through activation of cytotoxic T cells (CTLs) *via* interferon-γ (IFNγ) or tumor necrosis factor-α (TNF-α) (6). If viral particles and cellular components (either unmodified or chemically/genetically modified) are processed and presented to T cells, an autoimmune response is triggered, CTLs expand and directly kill cells or through Fas-ligand (FasL) secretion (10–13). Accumulating evidence has detailed the predominant role of CTLs in marrow destruction, and type I interferons (IFNs) polarizing the immune system toward Th1 responses (14–16); however, other T-cell subsets are involved, such as effector memory CD8^+^CD28^−^CD57^+^ T lymphocytes (17), CD4^+^CD25^high^FoxP3^+^ T regulatory cells (Tregs), and Th17 (18–20). IFN-γ and TNF-α are historically implicated in AA pathogenesis (7, 21). Despite great efforts in searching of the triggering viral agent, no conclusive results have been published, even though parvovirus B19 has been identified as the etiological cause of a subtype of pure red cell aplasia because erythroid precursors are the only permissive cell type for viral binding to host cell receptor (the P antigen) and replication (22).

### Large Granular Lymphocyte Leukemia

The LGL leukemia is classified as a lymphoproliferative disorder of mature T or natural killer (NK) cells and has an indolent course with cytopenia(s), recurrent infections, and splenomegaly (23). LGLs represent the neoplastic counterpart of effector memory and terminal effector memory CD8^+^ T cells with a characteristic skewing of the T-cell receptor (TCR) variable (V) α- and/or β-chain repertoire (24). Patients with LGL leukemia also display concomitant autoimmune disorders, especially rheumatoid arthritis or pure red cell aplasia (25, 26), and increased risk of B-cell neoplasia or MDS (27). In LGL leukemia, a chronic persistent antigen exposure is considered the initiating event by first inducing polyclonal CTL expansion and then an oligoclonal drift with the acquisition of additional pathologic hits, such as somatic mutations in *STAT3* or *STAT5b* and increased interleukin (IL)-15 and platelet-derived growth factor levels (24, 28, 29). Pathogenetic agents in LGL leukemia are still unknown, and the absence of recurrent TCR V α- or β-chain rearrangements among patients suggest that a common epitope might be missed (30–32).

### Paroxysmal Nocturnal Hemoglobinuria

Paroxysmal nocturnal hemoglobinuria, caused by somatic mutations in the X-linked phosphatidylinositol glycan class A (*PIGA*) gene involved in glycosylphosphatidylinositol (GPI) anchor biosynthesis (3), is characterized by failure of membrane anchorage of GPI-anchored proteins, including complement regulatory proteins, determining complement-mediated cell lysis that remains, to date, the only pharmacologically targeted pathway for the treatment of PNH (1, 5, 33). Several hypotheses have been made over decades from a proliferative advantage of GPI^−^ HSCs to a specific autologous immune response against GPI^+^ cells by CD1d-restricted T cells (8). The existence of a GPI-directed autologous T-cell clone is supported by the finding of highly homologous TCR β sequences among patients with PNH and AA, and also healthy subjects (34). Usually, clonotypes are private to each disease because of the unlimited number of possible epitopes in autoimmune diseases and cancers (17). The observation of recurrent PNH-related clonotypes in health and diseases suggests that common triggers might drive autoimmune responses against HSCs (17); however, it remains unclear why these autoreactive clones spare *PIGA* mutant cells that acquire a relative proliferative advantage over normal hemopoiesis.

In this study, we have tried to answer this unsolved question on whether BMF syndromes are triggered by infectious diseases or are caused by cross-reactivity between viral particles and cellular components.

## Methods, Definitions, and TCR Sequences

Epitope prediction is a complex bioinformatics procedure that requires appropriate software and tools, and sequences of individual TCR and matched major histocompatibility complex (MHC) class I type, not always available in clinical studies (35–37). In this study, we compared already published complementarity determining region 3 (CDR3) sequences expanded in BMF syndromes to repertoires reported in the literature for infectious diseases, such as cytomegalovirus (CMV) or Epstein–Barr virus (EBV), autoimmune disorders, and cancers. Lymphocytes are defined as oligoclonal when a random pool of T cells is represented by few clones (also termed as immunodominant clones or clonotypes) carrying a TCR with high antigen affinity, whereas T cells are polyclonal when recognizing a large number of epitopes (17). A clonotype is identified as immunodominant by flow cytometry Vβ usage analysis or by TCR Vα and/or Vβ repertoire next-generation sequencing (NGS) and is defined as the Vβ^+^ population(s) or the DNA sequence(s) present at the highest abundance (32). The oligoclonal expansion has been observed in PNH and AA and is the main pathogenetic alteration in LGL leukemia (30, 32, 38–43).

The CDR3 is the protein sequence included between the second conserved cysteine encoded by the 3′ region of Vβ segment and the conserved phenylalanine encoded by the 5′ portion of Jβ segment (44). We collected already reported CDR3 sequences in AA, PNH, or LGL leukemia, and immunodominant clones were selected (Supplementary Tables 1, 2) [(34) NCBI GEO accession no. GSE101660 from (17, 32, 45–48)]. Homology assessment was carried out by comparing CDR3 motifs to sequences present in the McPAS-TCR database (http://friedmanlab.weizmann.ac.il/McPAS-TCR/) containing TCR sequencing for known epitopes and diseases (49). The Max Levenshtein distance (edit distance between two sequences, i.e., sum of insertions, deletions, or substitutions) was first set on 0 (completely matched sequences) and then on 1 (sequences differed for one insertion, deletion, or substitution). Results were exported as csv files for further analysis or representation (Supplementary Table 3).

## Bone Marrow Failure-Related Clonotypes Are Frequently Associated With Infectious Diseases

For AA-related clonotypes, 13 out of 36 CDR3 motifs (36%) matched with known sequences in the database: only three (8%) exactly matched with other published sequences, and two of them were present in patients with AA with polyclonal CD8^+^ T cells, whereas one in a patient with the oligoclonal expansion of CD8^+^ effector memory T cells (Table 1). Matching or similar sequences were frequently associated with infectious diseases, such as *Mycobacterium tuberculosis* (eight matched sequences), CMV (*n* = 5), and influenza (*n* = 3), especially in patients with AA without effector memory T-cell oligoclonality. Matching AA-related clonotypes in patients with oligoclonal expansion (five total matches) were associated only with CDR3 motifs related to *M. tuberculosis* or CMV infection (*n* = 3 and *n* = 2, respectively), whereas AA-related clonotypes in patients with polyclonal expansion (eight total matches) were heterogeneously associated with infectious or autoimmune disorders and cancers. For PNH-related clonotypes, eight out of 12 sequences (66.7%) matched or had similarity with known CDR3 sequences recognizing the UL29/28 protein, a viral CMV component involved in activation of immediate-early gene expression. For LGL-related clonotypes, 20 out of 113 CDR3 motifs (17.7%) had similarities with reported sequences; 16 sequences were associated with motifs expanded in infectious diseases, such as *M. tuberculosis* (*n* = 9), influenza virus (*n* = 4), human immunodeficiency virus (HIV; *n* = 5), human T-cell lymphotropic virus type 1 (HTLV-1; *n* = 4), CMV (*n* = 3), or EBV. As controls, the three most expressed CDR3 sequences in healthy subjects (dataset GSE101660) were selected and used for homology assessment. Seven out of 27 sequences (26%) were found in the database and associated with CDR3 motifs described in infectious diseases, including *M. tuberculosis* (*n* = 3), and influenza virus (*n* = 4), or autoimmune disorders and cancers (*n* = 4) (Table 1 and Supplementary Figure 1A).

**Table 1:** Homology assessment between BMF-related clonotypes and public database.

**Disease**	**AA-clonotypes *N* = 36 (%)**	**PNH-clonotypes *N* = 12 (%)**	**LGL-clonotypes *N* = 113 (%)**	**HC-clonotypes *N* = 27 (%)**
CMV	5 (13.9%)	8 (66.7%)	3 (2.7%)	2 (7.4%)
*Mycobacterium* tuberculosis	8 (22.2%)	–	9 (8%)	3 (11.1%)
Influenza virus	3 (8.3%)	–	4 (3.5%)	4 (14.8%)
Yellow fever virus	1 (2.8%)	–	–	1 (3.7%)
HTLV-1	–	–	4 (3.5%)	1 (3.7%)
HIV	–	–	5 (4.4%)	–
EBV	–	–	1 (0.9%)	1 (3.7%)
Autoimmune diseases	2 (5.6%)	–	2 (1.8%)	1 (3.7%)
Cancers	2 (5.6%)	–	7 (6.2%)	3 (11.1%)

*BMF, bone marrow failure; AA, acquired aplastic anemia; PNH, paroxysmal nocturnal hemoglobinuria; LGL, large granular lymphocyte (leukemia); HC, healthy control; CMV, cytomegalovirus; HLTV-1, human T-lymphotropic virus type 1; HIV, human immunodeficiency virus; EBV, Epstein–Barr virus*.

## Bone Marrow Failure-Related Clonotypes Are Shared Between Health and Diseases

We have already described that CDR3 sequences of immunodominant clones in patients with AA and PNH are present at very low frequencies in healthy subjects (17). In particular, 32 out the 36 AA-clonotypes were found at low frequencies in the TCR repertoire of healthy subjects, whereas four to six PNH-clonotypes were present in AA and healthy subjects with the CATSRTGGETQYF motif observed in almost the entire cohort (17). Based on this initial observation, here we searched LGL-related sequences in AA and healthy TCR repertoire from the GSE101660 dataset. A total of 15 out of 113 LGL-related CDR3 motifs were recurrent in patients with AA. At least two sequences per AA patient were found in CD8^+^ T-cell TCR repertoire at very low frequencies (<0.005%) in subjects with oligoclonal expansion of effector memory CD8^+^ T cells, or at various levels (from <0.001 to 11.6%) in patients with polyclonal expansion. In a healthy TCR repertoire, a total of 17 out of 113 LGL-related motifs (15 in common with those found recurrent in AA) were observed at low frequencies (<0.014%) in healthy individuals (two to nine sequences per subject). To identify shared sequences between diseases and healthy subjects, we interpolated clonotypes selected in this analysis (Supplementary Figure 1B). Fourteen sequences were shared between patients with AA and LGL and healthy subjects, and one CDR3 motif between an AA subject (UPN, AA5) and patients with LGL leukemia. These sequences were associated with infectious diseases (*M. tuberculosis, n* = 6 matches; influenza virus, *n* = 3; CMV, *n* = 2; and HTLV-1/HIV, *n* = 2) or autoimmune disorders and cancers (*n* = 8).

## Discussion

**Figure 1:**
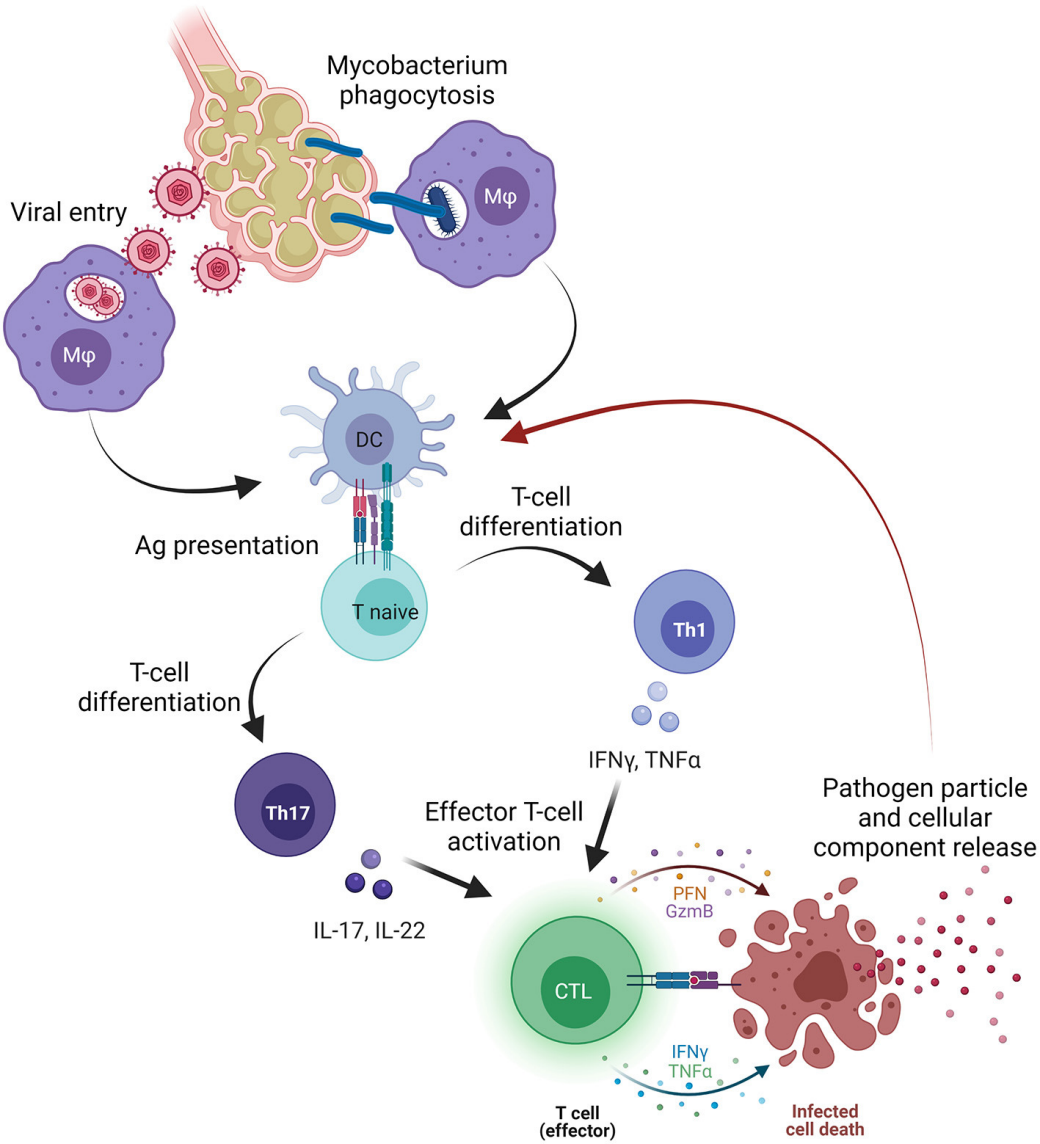
Pathogen involvement in bone marrow failure (BMF). Primary viral or mycobacterial infection occurs in the lower pulmonary system where pathogens infect alveolar cells and macrophages (Mφ), or where Mφ phagocyte mycobacteria containing the infection. Subsequently, Mφ migrate to pulmonary lymph nodes and presents antigens (Ag) to dendritic cells that processed and present Ag to naïve T cells. Once primed, naïve T cells differentiate primarily in T helper (Th) 1 cells releasing pro-inflammatory cytokines, such as tumor necrosis factor alpha (TNFα), interferon-gamma (IFNγ), or interleukins (IL) that, together with type I interferons (IFNs), trigger the activation of effector cytotoxic CD8+ T cells (CTLs). After activation, CTLs directly kill infected cells, and pathogen particles and cellular components are released and likely captured by dendritic cells, processed, and presented as novel antigens. On the other hand, pathogen particles might induce a cross-reactivity with cellular components and start an autoimmune response that might be sustained by Th17 cells and Th17-related cytokines (IL-17 or IL-22). The figure was created in Biorender.com.

Bone marrow failure-related clonotypes are considered private to each disease because of the unlimited number of possible epitopes (32, 50); however, why these clones share similarities with those associated with infectious diseases, especially *M. tuberculosis* and CMV infection, with this latter predominantly observed in PNH-related clonotypes, is still an open question (Figure 1).

Cytomegalovirus has a seroprevalence of 45–100% increasing with age. Primary infection occurs in most cases without symptoms, and CMV remains latent in CD34^+^ stem cells and CD33^+^ myeloid progenitors; however, only myeloid dendritic cells and monocytes are productively infected causing viral reactivation under immunosuppression, such as after transplantation and during IST (2, 51). In immunocompetent subjects, CMV reactivations are suppressed by killing infected cells, and chronic exposure to viral particles causes persistence of CMV-specific CD8^+^ T cells and selection of autoimmune clones (52). CMV might also shape the TCR repertoire of an individual and trigger CMV-specific clonotypes with low antigen affinity increasing the potential for autoimmune T-cell clone development (53). Indeed, about 10% of circulating CD8^+^ and CD4^+^ T cells are CMV-specific and persist in the blood suggesting a continuous stimulation by viral reactivation (52). After transplantation, CMV reactivation is associated with graft-vs.-host disease development underlying a possible cross-reactivity between CMV epitopes and some auto-(allo)-antigens, or an immune adjuvant function of CMV in the context of antigen presentation (2). Chronic release of GPI-anchored proteins might be a highly immunogenic stimulation for triggering autoimmune T-cell clones; indeed, studies on *Plasmodium* vaccines show that the combination of DNA vaccine with GPI-anchor signal sequences can dramatically enhance its immunogenicity (54). This may elicit the expansion of additional T-cell clones specific for these antigens expressed in normal hematopoietic cells, and eventually lacking on *PIGA*-mutant hematopoiesis. Therefore, at reactivation or in the presence of viral epitopes in the bone marrow, CMV-specific T cells may destroy not only infected CD34^+^ and CD33^+^ but also normal stem cells. At the same time, normal hematopoietic cells may be killed by additional cross-reactive autoimmune clones recognizing self-antigens (possibly GPI-linked) sparing *PIGA*-mutant hematopoietic cells lacking these surface self-motifs.

*Mycobacterium tuberculosis*, the pathogen responsible for the leading infectious cause of death worldwide, enters through the upper airways and arrives at the lower respiratory tract where is phagocyted by resident alveolar macrophages (55). The presence of intracellular bacterial materials can strongly induce a type I IFN response and macrophages can either go to apoptosis or necrosis (56). Infected macrophages, dendritic cells, or monocytes cross the alveolar epithelial barrier and migrate to pulmonary lymph nodes where prime antigen-presenting cells and trigger maturation of naïve CD4^+^ T cells (55). Following exposure, few subjects develop clinical symptoms and the disease, while the majority of individuals show resistance or early clearance of *M. tuberculosis*, are asymptomatic, or have a latent infection (56). IFNγ, likely secreted by CD4^+^ T cells, is important in controlling bacterial replication and resisters lack this IFNγ-mediated response (57, 58). Cytokines that interfere with or reduce IFNγ production, such as IL-10 or IL-17A, can contribute to the resister profile (59–61). TNFα has a protective role, and at low levels fails to contain immune response and favors the transition to active disease (55, 62). Moreover, TCR repertoire is different between resisters and latently infected patients, with various MHC-restricted αβ T cells recognizing a broad or limited repertoire of MHC class I and II-restricted *M. tuberculosis* peptides. This variability is likely related to a different immune response: Th1 cells are predominantly involved in latent infections; while unconventional MHC-independent T-cell responses, such as γδ or CD1-restricted T cells, are also activated in resisters (56, 63).

While reading mechanisms of infection and immune responses against CMV and *M. tuberculosis*, we might recall some features outlined in BMF pathogenesis, such as involvement of type I IFNs and TNFα and predominant roles of Th1 and CTLs (7); however, several questions might rise as well. First, we might wonder if what is observed in BMF is a common immune response against an insult, and BMF-related clonotypes just mirror an unspecific expansion of previously existing clones, or a physiologic T-cell response triggered by those pathogens might be somehow involved in the pathophysiology of BMF. We can only speculate that cross-reactivity between pathogen-related particles and cellular components expressed by hematopoietic cells may lead to an immune-mediated BMF, and differences in targeted epitopes and antigen-specific MHC class I or II-restricted T-cell responses might result in the wide clinical manifestation range of BMF syndromes. In the alternative, the postulated pathogenic role of CMV, *M. tuberculosis*, or other pathogens might be indirect, through a broad stimulation of T-cell response eventually leading to the emergence of pathogenic T-cell clones. We still do not know if pathogens are innocent bystanders because clonotypes are present at low frequency also in healthy individuals, suggesting that their association with PNH is not casual and that this could represent the starting point for mounting an autoimmune response against HSCs. These are open questions that need to be addressed; however, it might be worth not to search for “new friends,” while better investigate roles and involvements of old “foes” in marrow failure development.

## Data Availability Statement

The datasets presented in this study can be found in online repositories. The names of the repository/repositories and accession number(s) can be found in the article/Supplementary Material.

## Author Contributions

VG, AR, and CS conceptualized this study and wrote the manuscript. All authors reviewed the manuscript and agreed with the final version.

## Funding

This research was supported by the Intramural Program of the Department of Medicine, Surgery and Dentistry, University of Salerno, Italy.

## Conflict of Interest

The authors declare that the research was conducted in the absence of any commercial or financial relationships that could be construed as a potential conflict of interest.

## Publisher's Note

All claims expressed in this article are solely those of the authors and do not necessarily represent those of their affiliated organizations, or those of the publisher, the editors and the reviewers. Any product that may be evaluated in this article, or claim that may be made by its manufacturer, is not guaranteed or endorsed by the publisher.

## Supplementary Material

Supplementary Figure 1 **(A)** Homology assessment. Acquired aplastic anemia (AA)-, paroxysmal nocturnal hemoglobinuria (PNH)-, large granular lymphocyte (LGL) leukemia -, and healthy control (HC)-related clonotypes were used for homology assessment using the McPAS-TCR database, and the number of matches or similarities found in the database are reported based on the association of reported sequences with known diseases. CMV, cytomegalovirus; HLTV-1, human T-lymphotropic virus type 1; HIV, human immunodeficiency virus; EBV, Epstein–Barr virus. **(B)** Common sequences among bone marrow failure syndromes. VENNY tool (64) was used to find common or unique sequences from lists extrapolated from the TCR repertoire of HCs, patients with acquired AA, and large granular lymphocyte (LGL) leukemia.

Supplementary Table 1 CDR3 sequences for AA, PNH, and healthy subjects. CDR3, complementarity determining region 3; AA, acquired aplastic anemia; PNH, paroxysmal nocturnal hemoglobinuria.

Supplementary Table 2 CDR3 sequences for LGL leukemia. CDR3, complementarity determining region 3; LGL, large granular lymphocyte leukemia.
