# Supplementary figures and images for: Infectious Agents and Bone Marrow Failure: A Causal or a Casual Connection?
Source: Front Med (Lausanne). 2021 Nov 4;8:757730. doi: 10.3389/fmed.2021.757730 (PMC8599277; doi:10.3389/fmed.2021.757730)

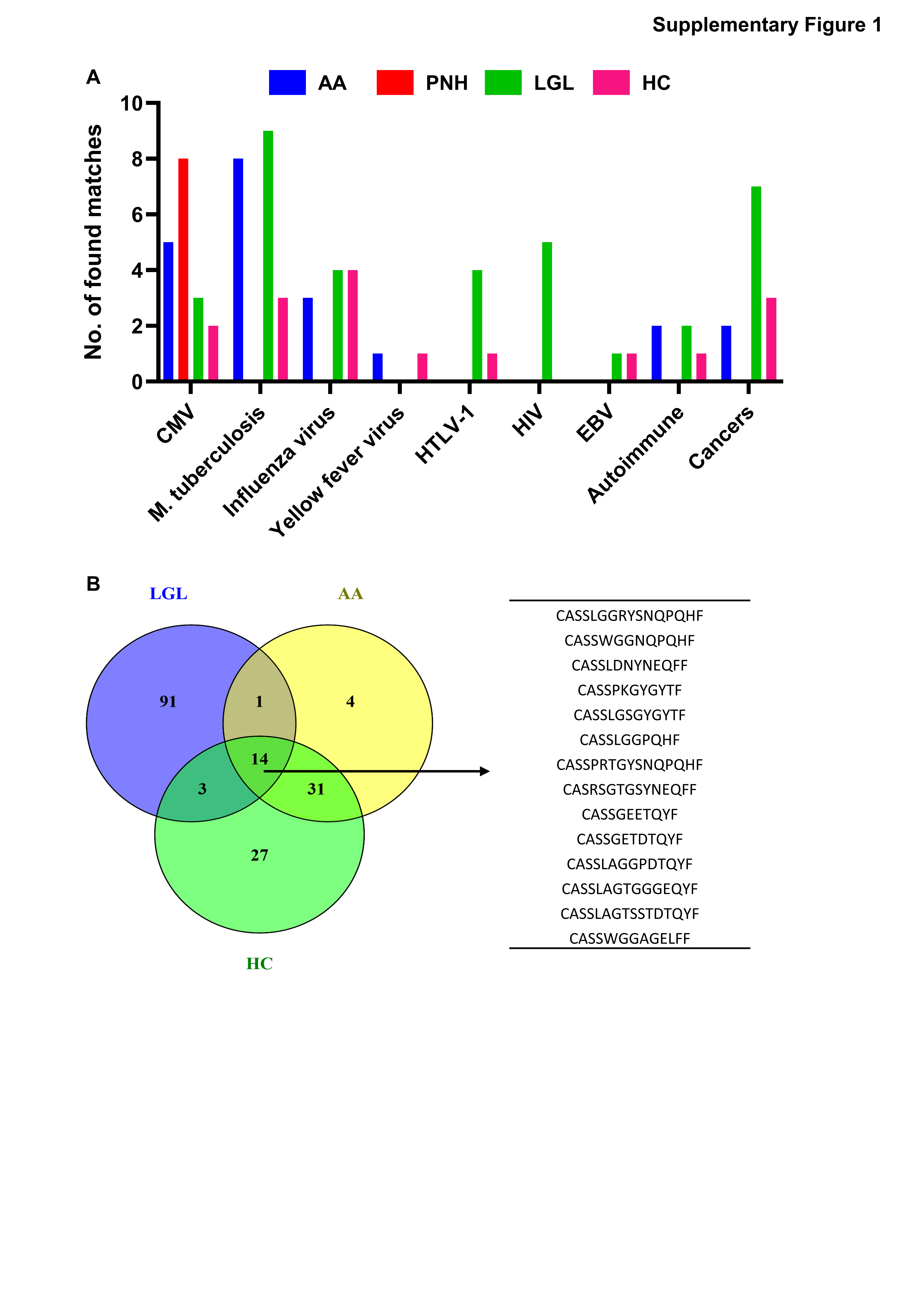

Supplement: Supplementary Figure 1 — (A) Homology assessment. Acquired aplastic anemia (AA)-, paroxysmal nocturnal hemoglobinuria (PNH)-, large granular lymphocyte (LGL) leukemia -, and healthy control (HC)-related clonotypes were used for homology assessment using the McPAS-TCR database, and the number of matches or similarities found in the database are reported based on the association of reported sequences with known diseases. CMV, cytomegalovirus; HLTV-1, human T-lymphotropic virus type 1; HIV, human immunodeficiency virus; EBV, Epstein–Barr virus. (B) Common sequences among bone marrow failure syndromes. VENNY tool (64) was used to find common or unique sequences from lists extrapolated from the TCR repertoire of HCs, patients with acquired AA, and large granular lymphocyte (LGL) leukemia. [file Image_1.TIF]
