# Supplementary material for: Infectious Agents and Bone Marrow Failure: A Causal or a Casual Connection?
Source: Front Med (Lausanne). 2021 Nov 4;8:757730. doi: 10.3389/fmed.2021.757730 (PMC8599277; doi:10.3389/fmed.2021.757730)
Supplement: Supplementary Table 1 — CDR3 sequences for AA, PNH, and healthy subjects. CDR3, complementarity determining region 3; AA, acquired aplastic anemia; PNH, paroxysmal nocturnal hemoglobinuria. [file Table_1.DOCX]

**Supplementary Table 1. CDR3 sequences for AA, PNH, and healthy subjects.**

| PNH | AA | Healthy subjects |
| --- | --- | --- |
| CASSLVGGPEQYF | CASSVRDYEQYF | CATSRDGDLGYNEQFF |
| CATSRGRTQGLDYGYTF | CSALPPGLASTDTQYF | CASSLDPGSYEQYF |
| CAWEQVIAFF | CASSQEVGGTNYGYTF | CATSLAG~DQPQHF |
| CATSGIAGETQFF | CATSTGTWTEQETQYF | CASSSANYGYTF |
| CATSRVAGETQYF | CASSFRDWGRYEQYF | CSAPGGGGQGNPEQYF |
| CATSRIGGETQYF | CSARDLAEEQYF | CASSQEQTDANTEAFF |
| CATSRTAGETQYF | CSARDPPVSGTRGTDTQYF | CASSLDSPPFGELFF |
| CATSRTGGETQYF | CASSYRETNEQFF | CSVEGGSSYEQYF |
| CATSRIAGETQYF | CATSRETGAAEQYF | CASMDWGQDRAYEQYF |
| CATSRVGGETQYF | CATSSSRSGQGLNEQFF | CAISELWARPVGNSPLHF |
| CATSREIGETQYF | CATSRPFPGQGAN*AEAFF | CASQGTGEKTQYF |
| CATSRDLAGETQYF | CATSDPLTASYEQYF | CASSERKG~GGSTEAFF |
|  | CASGGANSPLHF | CASSGGGRGIKNEQYF |
|  | CSAPDDGANVLTF | CASSLEGGYTDTQYF |
|  | CSVEADNRAGANVLTF | CSAIPTGTYEQYF |
|  | CASSLD~SNEQFF | CASSALAGAGDTQYF |
|  | CASSEGLESETQYF | CASTID~GELFF |
|  | CSAPGSGDRNEQFF | CASSDSSGGNSPLHF |
|  | CASSFTGELFF | CASSLAERLSSYNEQFF |
|  | CATGSTRTGGRTEAFF | CASSTWDRGSRETQYF |
|  | CASSLAGNYGYTF | CSAVRQGEYGYTF |
|  | CSARDAADYEQYF | CASSIVGRGDTEAFF |
|  | CSASRAGGVTEAFF | CSASGASGELRETQYF |
|  | CSARDAP~GANVLTF | CSVEVSWTGG*ETQYF |
|  | CSATDG~NQPQHF | CASSLGETQYF |
|  | CATSRVAGETQYF | CSSGPYNEQFF |
|  | CASSSPGQFDEQYF | CASSLAPGSTYEQYF |
|  | CASSLAGGPDTQYF |  |
|  | CASGDGGTDTQYF |  |
|  | CASSLDLTGNTEAFF |  |
|  | CAISESSFSSQETQYF |  |
|  | CASSESRGYNYGYTF |  |
|  | CASSQDLLPGDNSPLHF |  |
|  | CASSQR~GGYTF |  |
|  | CASSDGGPSYEQYF |  |
|  | CASSPRQGVDEQYF |  |

**Abbreviations.** CDR3, complementarity determining region 3; AA, acquired aplastic anemia; PNH, paroxysmal nocturnal hemoglobinuria.
