# Supplementary material for: Infectious Agents and Bone Marrow Failure: A Causal or a Casual Connection?
Source: Front Med (Lausanne). 2021 Nov 4;8:757730. doi: 10.3389/fmed.2021.757730 (PMC8599277; doi:10.3389/fmed.2021.757730)
Supplement: Supplementary Table 2 — CDR3 sequences for LGL leukemia. CDR3, complementarity determining region 3; LGL, large granular lymphocyte leukemia. [file Table_2.DOCX]

**Supplementary Table 2. CDR3 sequences for LGL leukemia**

| LGL sequences | | |
| --- | --- | --- |
| CAWPD*REYNE | CASSLPGTPTE | CASSGEETQYF |
| CATSGTGVPHYNE | CASSLGGPQHF | CASSGETDTQYF |
| CASSIGIQP | CASSLASISYNE | CASSLAGGPDTQYF |
| CASSLIGVSSYNE | CASSLEYRVGE | CASSLAGTGGGEQYF |
| CASSIVAAHYG | CASSAGQDTE | CASSLAGTSSTDTQYF |
| CASKSGDPGE | CASSPHGVTVSLENTE | CASSLGSYNSPQHFDG |
| CASSVGRFQET | CASARSHGLLRAV | CASSLGSYNSPLH |
| CAWSPNTE | CAWSEPPLLDVSGSYNE | CASSLLKPTSDTQYF |
| CASSLLAGGYNE | CASSLLTKTGSYEQE | CASSLLTKTGSYEQYF |
| CASSSLGVPYYE | CAISGTGILPG | CASSLVGDPGELF |
| CASSVGQGSP | CSASGLLAGGPSYE | CASSLVGGRPSYNEQFF |
| CASSLVPGTLNTGE | CATSRDMGEQE | CASSPPVRARDTQYF |
| CASSRSGWSSDSP | CASSHGGLDE | CASSPPVRSRAQY |
| CASSQGRG | CSAQTGRYEQE | CASSWGGAGELFF |
| CASSLSGRALNEQFF | CSASPLWTGNTE | CASSWGGRGEQFF |
| CAISEGSGPGELFF | CASSFRQNNE | CATSDLGQGASGELF |
| CASGGDRGTGANVLTF | CASSIDRGRRETQ | CATSDLGQQETQY |
| CASSLGGRYSNQPQHF | CASSPLGAVGYNE | CATSDLGEKVGNEQFF |
| CSASLGGRPTIAGNTIYF | CASSLFVQPPLDIPYNS | CATSRDLAGEKLF |
| CASSQDVRPPPEDRPYSNQPQHF | CASSSGPTQGVLDISETE | CATSRDLASGRETQYF |
| CASSLGTGGMETQYF | CASSPTLRDRGRTDT | CATSRDLRGEKLF |
| CASSLAHSYNEQFF | CASSPPVGDRSYE | CATSRDLRTQETQY |
| CGSLGQGAWYEQYF | CASSAGLNG | CASRDRAGN |
| CASSPESLFSYEQYF | CASSYLLTTGGAVRS | CASRSGTGAHEQYF |
| CARSFSPSLDTSSLFVEAFF | CASTWGGNGYE | CASRSGTGSYNEQFF |
| CASSCYQPGLDLPRADTQYF | CASSSLRSGPMNTEAFF | CASRQPSYE |
| CASSIFRGNEQYF | CASSLAWGINSPLHF | CASISAVRLASTDT |
| CASSPVGAYPKETQYF | CASSLRAGGPNEQFF | CASSPGTGRNQPQHFGD |
| CASSLAKGKGATNEKLFF | CSASDRNSNQPQHF | CASRDGAGEQYF |
| CASNNRGSYNEQFF | CASSRTGILAKNIQYF | CASSLGSGYGYTF |
| CASSWGGNQPQHF | CASSQLDRVYGANVLTF | CASGQRGGA |
| CAISGRLAGGRTSTDTQYF | CASSQTSGGEYNEQFF | CASSYTFQGETQY |
| CASGGVGGGELFF | CASSPRTGYSNQPQHF | CASSPGTGLDGYTF |
| CASSLDNYNEQFF | CASSPLGLETQYF | CASSYGSPLDIDSAISPQHF |
| CASSFSPYTRPEAFF | CASSVPGGEQYF | CASSPKGYGYTF |
| CAVPTGRNEKLFF | CASSVPGGEQYF | CASSYYREPQHFGD |
| CATSRDLLTYEQYF | CASSVPGGEQYF | CASSYTFQGETQY |
| CSARNGPNYQETQYF | CASSYYREPQHFGD |  |

**Abbreviations.** CDR3, complementarity determining region 3; LGL, large granular lymphocyte leukemia.
